# Selenitetriglyceride-Induced Modulation of Selected Cellular and Humoral Immune Parameters in Kamieniecka Sheep

**DOI:** 10.3390/ani15233362

**Published:** 2025-11-21

**Authors:** Bartosz Orzechowski, Jan Miciński, Katarzyna Ząbek, Grzegorz Zwierzchowski, Roman Wójcik

**Affiliations:** 1 Department of Sheep and Goat Breeding, Faculty of Animal Bioengineering, University of Warmia and Mazury in Olsztyn, Oczapowskiego 5, 10-917 Olsztyn, Poland; bartosz.orzechowski@student.uwm.edu.pl (B.O.); micinsk@uwm.edu.pl (J.M.); katarzyna.zabek@uwm.edu.pl (K.Z.); 2 Department of Biochemistry, Faculty of Biology and Biotechnology, University of Warmia and Mazury in Olsztyn, Oczapowskiego 1A, 10-719 Olsztyn, Poland; grzegorz.zwierzchowski@uwm.edu.pl; 3 Department of Microbiology and Clinical Immunology, Faculty of Veterinary Medicine, University of Warmia and Mazury in Olsztyn, Oczapowskiego 13, 10-718 Olsztyn, Poland

**Keywords:** Kamieniecka sheep, selenitetriglycerides, proliferative activity of blood lymphocytes, phagocytic activity of monocytes and granulocytes, gamma globulin levels, ceruloplasmin activity, lysozyme activity

## Abstract

With the aim to investigate the immunomodulatory potential of selenitetriglycerides (SeTG), a new lipophilic Se (IV) compound, 30 sheep (15 sheep/treatment) were used in a completely random design to receive the SeGT supplement as follows: (1) no SeGT supplement (Control) and (2) daily dosage of 2 mL of SeGT (equivalent to 1 mg Se/kg BW) during the first 7 days of the evaluation, which lasted 28 d. Individually, blood samples were collected on days 0, 14, and 28 to measure and assess parameters of innate cellular and humoral immunity, including respiratory burst activity (RBA) and potential killing activity (PKA) of monocytes and granulocytes, proliferative response of lymphocytes stimulated with ConA (Concovalin A) and LPS (lipopolisaccharidde), lysozyme activity, ceruloplasmin activity, and gamma globulin levels. From the 14th day, supplemental SeTG saw significant increases (*p* ≤ 0.001) in RBA and PKA parameters, as well as enhanced proliferative responses of lymphocytes compared with controls. Both innate humoral immunity (elevated lysozyme activity) and adaptive humoral immunity (increased gamma globulin levels) were positively influenced (*p* ≤ 0.01), whereas ceruloplasmin activity remained unchanged. Under the conditions in which the current experiment was carried out, SeGT showed good promise to modulate immunity in a short period (28 d). Further research should explore experiments with a greater number of animals over long-term periods of evaluation under production system conditions.

## Simple Summary

Selenium is a natural mineral that animals require in small amounts to remain healthy, particularly for maintaining a strong immune system. When animals do not receive enough selenium, they become more susceptible to infections and other health problems. Farmers typically provide selenium to livestock in the form of salt or yeast, but new types of selenium supplements are being developed to be more effective and safer. In this study, we tested a new form of selenium, selenitetriglyceride, in sheep. We collected blood samples and assessed the functioning of both innate and adaptive immune responses. The results showed that sheep given this supplement had stronger immunity: their white blood cells were more effective at fighting germs, natural protective substances in the blood, such as lysozyme, were higher, and the levels of gamma globulins, which indicate specific antibodies, also increased. Importantly, the supplement did not cause any adverse effects. These findings suggest that this new type of selenium could be a valuable tool for farmers to keep sheep healthier and less prone to illness. Stronger immunity in farm animals leads to better animal welfare, fewer losses for farmers, and potentially less need for antibiotics, which benefits both agriculture and society.

## 1. Introduction

Selenium (Se) is an essential trace element involved in numerous physiological processes that contribute to the maintenance of health and immune homeostasis in both humans and animals. Its well-established functions include antioxidant activity, detoxification, thyroid hormone regulation, and immune modulation [1,2,3]. In livestock, selenium is also vital for reproductive performance, neonatal development, and disease resistance. Deficiency has been associated with conditions such as nutritional muscular dystrophy, impaired fertility, placental retention, and increased susceptibility to infections [4,5,6,7]. Additionally, selenium imbalance can negatively affect growth, milk yield, and overall productivity, highlighting its importance for both animal welfare and farm economics [1,4].

Assessment of selenium status in animals is typically based on blood selenium concentration or the enzymatic activity of glutathione peroxidase (GSH-Px) in erythrocytes, both serving as reliable biomarkers of selenium availability and utilisation [8,9]. Beyond these markers, the impact of selenium on immune system function is receiving increasing attention. Selenium has been shown to influence lymphocyte proliferation, immunoglobulin production, neutrophil activity, and cytokine expression, thereby supporting both cellular and humoral immune responses, including innate and adaptive immunity [2,10,11]. Moreover, Se contributes to the regulation of redox-sensitive signalling pathways, modulation of inflammation, and antiviral defence mechanisms, which are particularly important in ruminant health management [12].

Conventional selenium supplementation in livestock diets relies on inorganic (e.g., sodium selenite) or organic (e.g., selenomethionine) forms, but recent efforts have focused on novel, more bioavailable and less toxic selenium compounds. Organic forms are generally associated with higher absorption and retention, while inorganic salts, although cost-effective, may have limited efficacy and higher pro-oxidative potential [1,4]. One such innovation is the development of selenitetriglycerides (SeTG)—lipophilic organoselenium compounds synthesised through esterification of selenic acid with oxidised triglycerides [13]. Characterised by selenium in the +4-oxidation state, these compounds demonstrate improved tissue distribution and reduced toxicity compared to sodium selenate [14]. Animal studies have confirmed their high tolerance levels and preferential accumulation in the liver and kidneys, with rapid excretion primarily via the urinary route [15].

Despite promising pharmacokinetic and toxicological profiles observed in rodent models, the use of SeTG in ruminants remains largely unexplored. Very limited data exists regarding their immunomodulatory effects, particularly in relation to immune responses [16]. Given the increasing demand for more effective and safer supplementation strategies in animal production, there is a clear need to investigate the potential of such compounds in enhancing immune resilience without compromising animal welfare. Furthermore, with growing restrictions on antibiotic use in food-producing animals, nutritional immunomodulators such as selenium derivatives may play an important role in improving disease resistance and supporting sustainable livestock systems [17,18].

The objective of the present study was to evaluate the effects of dietary supplementation with SeTG on selected innate immune parameters in Kamieniecka sheep. Specifically, we assessed lymphocyte proliferation, phagocytic activity of peripheral blood monocytes and granulocytes, and selected indicators of both innate and adaptive humoral immunity. This study aims to contribute to the growing body of knowledge on novel selenium formulations in ruminant nutrition and to assess their potential as functional feed additives with immunomodulatory properties.

## 2. Materials and Methods

### 2.1. Animal and Experimental Design

The study was carried out with the approval of the Local Ethics Commission for Animal Experiments (approval no. 34/2021, issued on 19 May 2021). It involved 30 female Kamieniec sheep maintained at the Komalwy breeding farm in the Warmia-Mazury Voivodeship, Poland. The animals were selected by the analogue method from a base herd of 350 breeding ewes. All sheep were three years old, with an average body weight of 57 kg (ranging from 55 to 60 kg), and were three months post-lambing. Prior to selection, all individuals were examined by ultrasound (ForVet, Dramiński, Sząbruk, Poland) to confirm the absence of pregnancy, and none were found to be pregnant. The animals were housed year-round in a free-stall system on deep straw bedding, under conditions compliant with the welfare standards for farm animals [19]. In brief, the experimental animals were kept in an indoor sheepfold, where individual pens of 2.5 m^2^ per animal were provided. Sheep had access to single-sided feeders arranged along the barn, with 0.8 m of feeder space per animal. Each pen was equipped with an individual bowl drinker (La Buvete, Paris, France) made of cast iron with a valve activated by a pressure of 500 g, allowing free access to water with a flow rate of up to 8 L/min. The indoor temperature ranged from 12 to 15 °C, and air volume per animal was 4.7 m^3^. Natural gravity ventilation maintained an air velocity of approximately 0.3 m/s.

All sheep were fed the same diet in the form of a total mixed ration (TMR) using the ad libitum feeding method. The TMR consisted of grass silage (45%), maize silage (30%), hay (20%), concentrate mix (4.5%), and a mineral and Milafos L vitamin mix (0.5%) (GrainCrop Animal Nutrition, Victoria, Austrilia). The concentrate mixture included oat (50%), wheat (30%), maize (10%), and soya (10%) meals. All feeds were balanced according to established nutritional standards established by INRA/INRAE [20] and adjusted to the physiological requirements of ewes at this stage of production. On average, each sheep consumed per day: 2.8 kg of grass and maize silage, 0.6 kg of meadow hay, and 0.6 kg of concentrate mixture.

The sheep were randomly assigned to two groups: a control group (C) and a SeTG-supplemented group (S), each consisting of 15 animals. Animals in the supplemented group received SeTG, while the control group received no supplementation. The supplemented group received a daily dose of 2 mL SeTG per animal for seven consecutive days (equivalent to 1 mg Se/kg body weight), while the control group received 2 mL of distilled water as a placebo, starting from day 0. The selected dose was determined based on previous experimental studies conducted in rats [21] and sheep [22]. The liquid preparation was administered orally each morning using a calibrated dripper.

### 2.2. Blood Sample Collection and Analysis

Blood samples were obtained from all experimental animals from the external jugular vein of sheep on day 0 and subsequently on days 14 and 28 of the trial. The collected material was aliquoted into tubes containing a clot activator (9 mL, Vacuette, Greiner Bio-One, Kremsmünster, Austria) for the evaluation of selected indicators of humoral immunity, and into tubes containing lithium heparin (6 mL, Vacuette, Greiner Bio-One, Kremsmünster, Austria) for the assessment of cellular immune parameters. Samples designated for the determination of lysozyme activity and ceruloplasmin concentration (innate humoral immunity), as well as gamma globulin levels (adaptive humoral immunity), were stored at −20 °C until analysis, whereas the remaining assays were performed within 3 h of collection. 

### 2.3. Evaluation of Non-Specific Cellular Immunity Parameters

The metabolic activity of phagocytic cells in blood leukocytes was evaluated using assays designed to assess specific intracellular functions. Respiratory burst activity (RBA) was measured after stimulation with phorbol myristate acetate (PMA; Sigma-Aldrich, Bellefonte, PA, USA) according to the method described by Chung and Secombes [23], as modified by Siwicki et al. [24]. Potential killing activity (PKA) of both mononuclear (MN) and polymorphonuclear (PMN) phagocytes was determined after incubation with inactivated microorganisms, following the procedure of Rook et al. [25], adapted by Siwicki et al. [24]. Lymphocyte proliferation was quantified using the MTT colorimetric assay after stimulation with concanavalin A (ConA) or lipopolysaccharide (LPS), following the original approach described by Mosmann [26] and refined by Wagner et al. [27].

### 2.4. Evaluation of Specific and Non-Specific Humoral Immunity Parameters

The serum gamma-globulin concentration was determined using the precipitation method modified by Siwicki and Anderson [28]. Plasma lysozyme activity was assessed by the turbidimetric technique [29] also adapted by Siwicki and Anderson [28]. Ceruloplasmin activity in plasma was determined according to the procedure established by Siwicki and Studnicka [30].

### 2.5. Statistical Analysis

Data were systematically organised in Excel spreadsheets for subsequent statistical analysis. The normality of distributions and the homogeneity of variances were assessed using the Shapiro–Wilk and Levene tests, respectively. Results were expressed as arithmetic means ± standard deviation (SD). Differences were evaluated using two-way analysis of variance (ANOVA) with the factors “group” (control vs. experimental) and “time” (days 0, 14, 28). When significant main or interaction effects were observed, pairwise comparisons were performed using Bonferroni-corrected *t*-tests. Statistical significance was indicated as follows: * *p* < 0.05; ** *p* < 0.01; *** *p* < 0.001; **** *p* < 0.0001. Results presented in tables were reported to three decimal places. All statistical analyses were performed using GraphPad Prism software, version 10.6.1.

## 3. Results

### 3.1. Non-Specific Cellular Immunity Parameters

**Figure 1:**
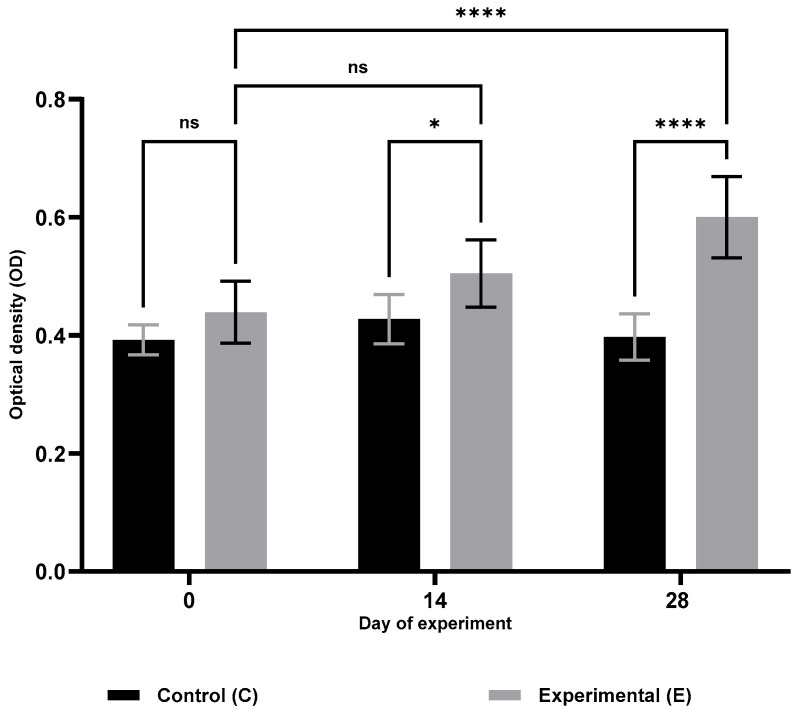
Changes in respiratory burst activity (RBA) of blood monocytes and granulocytes in sheep following selenitetriglycerides supplementation. Bars represent mean optical density (OD) values measured at 620 nm ± SD. Control group (C), black bars; Experimental group (E), grey bars. Statistical differences were evaluated using two-way ANOVA followed by Bonferroni-corrected pairwise *t*-tests. Significance levels: ns, not significant; *p* < 0.05 (*); *p* < 0.0001 (****).

**Figure 2:**
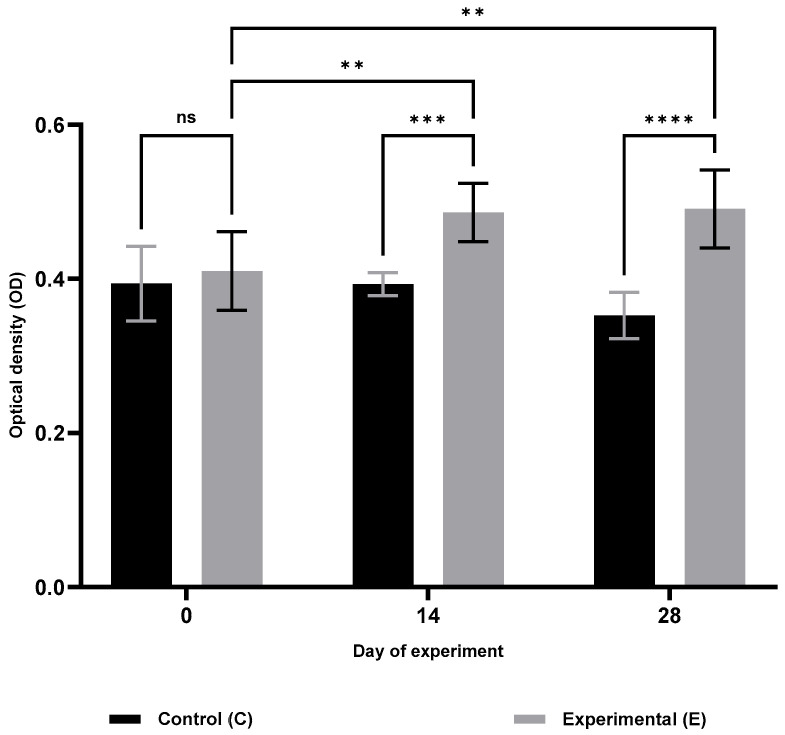
Changes in potential killing activity (PKA) of blood monocytes and granulocytes in sheep after dietary supplementation with selenitetriglycerides. Bars represent mean optical density (OD) values measured at 620 nm ± SD. Control group (C), black bars; Experimental group (E), grey bars. Statistical differences were evaluated using two-way ANOVA followed by Bonferroni-corrected pairwise *t*-tests. Significance levels: ns, not significant; *p* < 0.01 (**); *p* < 0.001 (***); *p* < 0.0001 (****).

**Figure 3:**
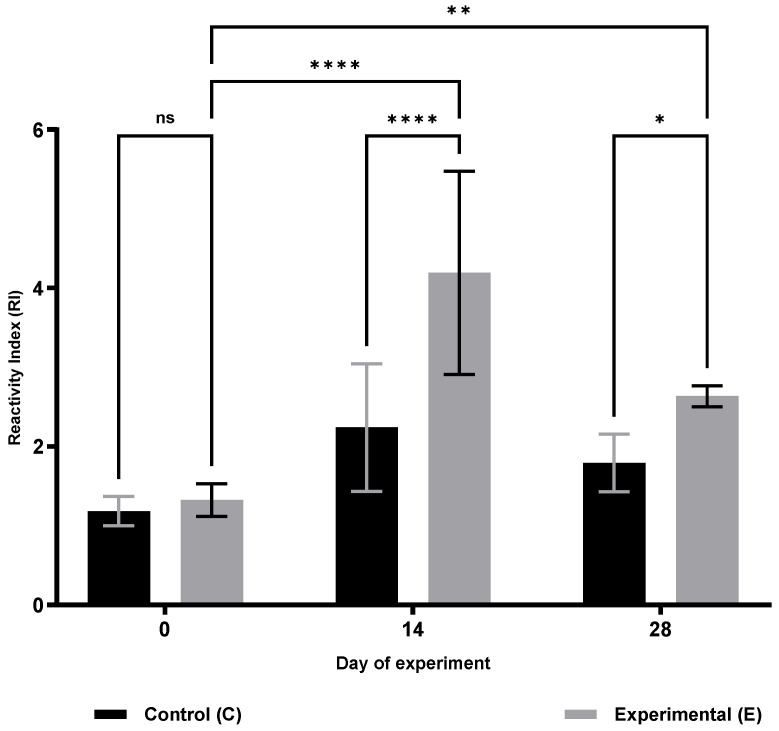
Changes in the proliferative response of ConA-stimulated lymphocytes (T lymphocytes) in sheep after dietary supplementation with selenitetriglycerides. Bars represent mean optical density (OD) values measured at 620 nm ± SD. Control group (C), black bars; Experimental group (E), grey bars. Statistical differences were evaluated using two-way ANOVA followed by Bonferroni-corrected pairwise *t*-tests. Significance levels: ns, not significant; *p* < 0.05 (*); *p* < 0.01 (**); *p* < 0.0001 (****).

**Figure 4:**
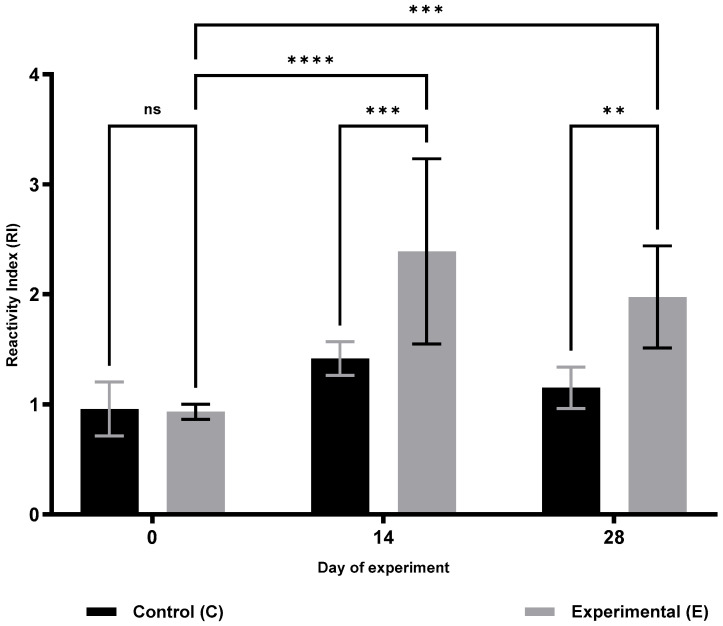
Changes in the proliferative response of LPS-stimulated lymphocytes (B lymphocytes) in sheep after dietary supplementation with selenitetriglycerides. Bars represent mean optical density (OD) values measured at 620 nm ± SD. Control group (C), black bars; Experimental group (E), grey bars. Statistical differences were evaluated using two-way ANOVA followed by Bonferroni-corrected pairwise *t*-tests. Significance levels: ns, not significant; *p* < 0.01 (**); *p* < 0.001 (***); *p* < 0.0001 (****).

Analysis of cellular immunity parameters (Table 1) revealed a significant increase in respiratory burst activity (RBA; Figure 1) and potential killing activity (PKA; Figure 2) in the supplemented group (S) on days 14 and 28 of the trial (RBA: *p* ≤ 0.05 and *p* ≤ 0.0001; PKA: *p* ≤ 0.001 and *p* ≤ 0.0001, respectively). Furthermore, lymphocyte proliferation in response to lipopolysaccharide (B lymphocytes; MTT LB, Figure 3) and ConA stimulation (T lymphocytes; MTT LT, Figure 4) was significantly enhanced on both days 14 and 28 compared with the control group (C) (MTT LB: *p* ≤ 0.001 and *p* ≤ 0.01; MTT LT: *p* ≤ 0.0001 and *p* ≤ 0.05, respectively). Relative to baseline (day 0), significant increases were detected in group E on day 28 for all assessed cellular parameters: RBA (*p* ≤ 0.0001), PKA (*p* ≤ 0.01), MTT LB (*p* ≤ 0.001), and MTT LT (*p* ≤ 0.01).

### 3.2. Specific and Non-Specific Humoral Immunity Parameters

**Figure 5:**
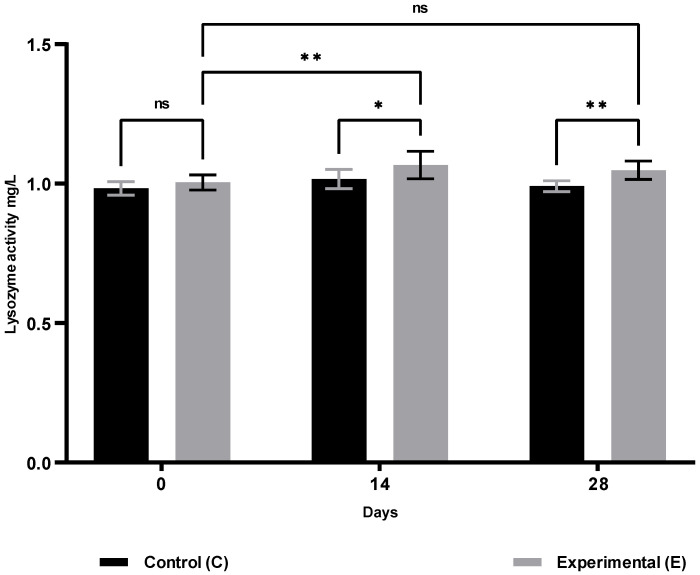
Lysozyme activity in sheep blood serum in the supplemented group (selenitetriglycerides supplementation) and control group. Bars represent mean optical density (OD) values measured at 620 nm ± SD. Control group (C), black bars; Experimental group (E), grey bars. Statistical differences were evaluated using two-way ANOVA followed by Bonferroni-corrected pairwise *t*-tests. Significance levels: ns, not significant; *p* < 0.05 (*); *p* < 0.01 (**).

**Figure 6:**
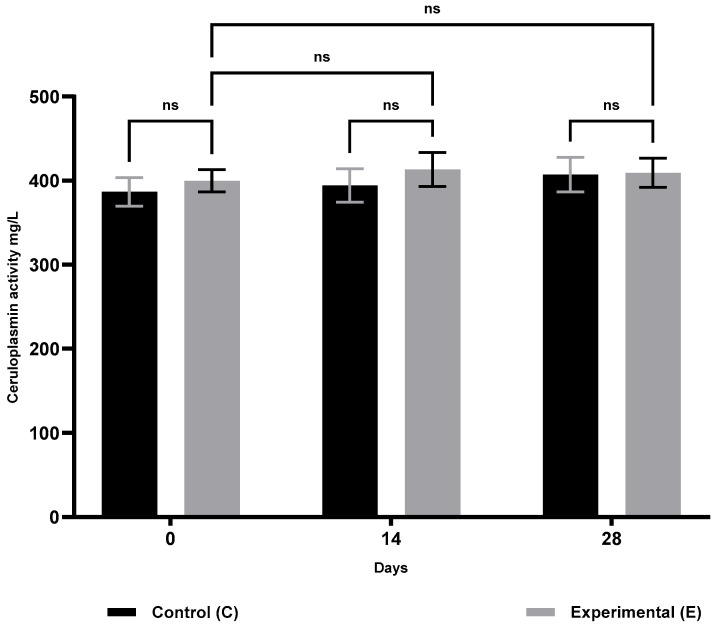
Ceruloplasmin activity in sheep blood serum in the supplemented group (selenitetriglycerides supplementation) and control group. Bars represent mean optical density (OD) values measured at 620 nm ± SD. Control group (C), black bars; Experimental group (E), grey bars. Statistical differences were evaluated using two-way ANOVA followed by Bonferroni-corrected pairwise *t*-tests. Significance levels: ns, not significant.

**Figure 7:**
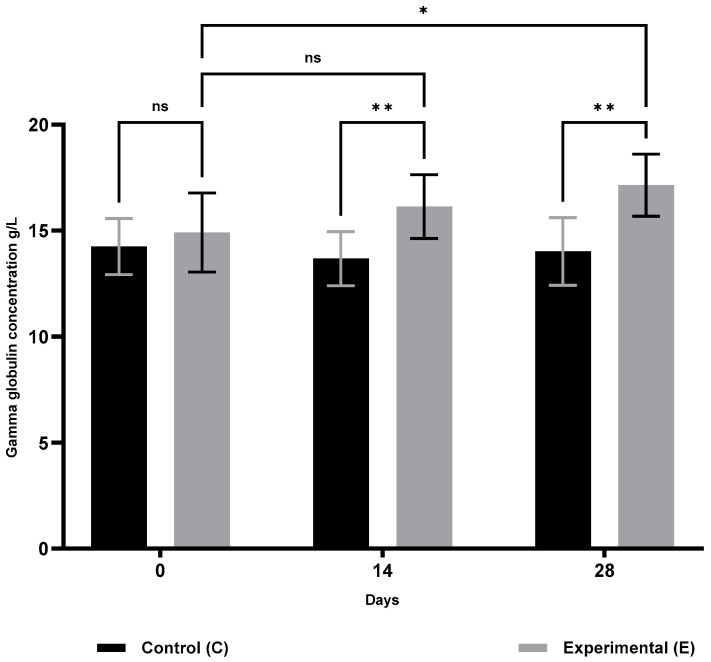
Gamma globulin concentrations in sheep blood serum in the supplemented group (selenitetriglycerides supplementation) and control group. Bars represent mean optical density (OD) values measured at 620 nm ± SD. Control group (C), black bars; Experimental group (E), grey bars. Statistical differences were evaluated using two-way ANOVA followed by Bonferroni-corrected pairwise *t*-tests. Significance levels: ns, not significant; *p* < 0.05 (*); *p* < 0.01 (**).

The results of humoral immunity are presented in Table 2. Compared with the control group, animals in group S showed a significant increase in lysozyme activity (Figure 5) on days 14 (*p* ≤ 0.05) and 28 (*p* ≤ 0.01), and in gamma globulin concentrations (Figure 6) on days 14 and 28 (*p* ≤ 0.01). No significant changes were observed in ceruloplasmin activity (Figure 7) throughout the study period. Compared with baseline (day 0), group S showed a significant increase in lysozyme activity only on day 14 (*p* ≤ 0.01), while gamma globulin levels, reflecting adaptive humoral immunity, increased significantly only on day 28 (*p* ≤ 0.05).

## 4. Discussion

This study presents new evidence on the immunomodulatory role of SeTG, a novel lipophilic selenium compound, in sheep. Supplementation enhanced respiratory burst activity (RBA) and potential killing activity (PKA) of phagocytes, increased lymphocyte proliferation, elevated lysozyme activity (innate humoral immunity), and raised gamma globulin concentrations (adaptive humoral immunity), while ceruloplasmin activity remained unchanged. These outcomes are consistent with a broad range of previous findings confirming that selenium is a key micronutrient in the regulation of both innate and adaptive immunity.

The increase in phagocyte functions observed in our experiment reflects the central role of selenium in supporting oxidative metabolism and microbicidal activity. Selenium-dependent selenoproteins such as glutathione peroxidase and thioredoxin reductase protect neutrophils from oxidative damage during reactive oxygen species (ROS) production, thereby sustaining pathogen elimination [31,32,33]. Hall et al. [34] reported that selenium supplementation improved neutrophil function in cattle, while Milewski et al. [35] observed that selenium supplementation enhanced oxidative burst in lamb neutrophils. Weiss and Hogan [36] described improved neutrophil bactericidal efficiency in dairy cows after parenteral selenium, and Hogan et al. [37] reported enhanced ROS production in dairy cows. Similar effects were documented in dairy cows and pigs, where selenium supplementation, including selenium yeast, improved neutrophil bactericidal activity [38], and in poultry, where selenium supplementation enhanced the phagocytic and oxidative capacity of macrophages [39,40,41]. Studies in goats also demonstrated increased leukocyte oxidative activity following selenium administration [6]. These findings collectively support our observation that SeTG act in line with established selenium sources to enhance innate cellular immunity, particularly neutrophil-dependent defences.

The stimulation of lymphocyte proliferation in response to both ConA and LPS further indicates that SeTG support adaptive cellular responses. Selenium deficiency is associated with reduced lymphocyte blastogenesis, impaired IL-2 synthesis, and weaker T-cell signalling [33,42,43]. Kumar et al. [44] showed that selenium supplementation increased mitogen-induced lymphocyte activity in lambs, while Hall et al. [16] confirmed similar improvements in calves. Other studies have reported enhanced lymphocyte transformation rates in selenium-supplemented calves [45] and increased lymphocyte proliferation in pigs [46,47]. Rodent studies have further confirmed that selenium depletion reduces T-cell responses, while repletion restores proliferative capacity [48]. These effects are mediated by selenium’s role in regulating transcription factors such as NF-κB and AP-1, which are essential for cytokine expression and lymphocyte expansion [49,50]. Thus, our findings reinforce the broader evidence that selenium promotes both T- and B-cell activation, with SeTG producing similar immunostimulatory effects.

Humoral immune parameters also strongly support selenium’s role in immunity. Lysozyme, a key component of innate humoral defence, was significantly increased in the supplemented group. Similar effects were reported in lambs supplemented with selenium [35] and in sheep given long-acting selenium preparations [10]. Fish studies have shown that extended dietary supplementation with organic selenium (Se-yeast) can enhance lysozyme activity and support innate immune function [51]. In laying hens, supplementation with selenium-enriched earthworm powder increased serum lysozyme, immunoglobulins, and antioxidant enzyme activity [52]. Comparable benefits were reported in marine fish (*Argyrosomus regius*), where selenium yeast enhanced both lysozyme activity and other innate immune enzymes [53]. In contrast, ceruloplasmin activity remained unaffected, which agrees with earlier reports suggesting that this copper-dependent acute phase protein is more strongly influenced by copper status and inflammation than by selenium supplementation [54,55]. These findings highlight that ceruloplasmin is not a sensitive biomarker of selenium-related immune modulation in ruminants.

In addition to innate humoral markers, adaptive humoral immunity was also enhanced, as evidenced by a significant increase in gamma-globulin concentrations in the supplemented group. Gamma globulins mainly comprise immunoglobulins (IgG, IgM, IgA), which play a crucial role in acquired immunity by neutralising pathogens. The immunostimulatory effects of selenium on antibody production have been widely documented in various livestock species. In poultry, particularly when using organic or nanoparticle forms, selenium has been shown to enhance antibody responses to antigens and vaccines. For example, Mohammadi et al. [56] demonstrated that broilers supplemented with nano-selenium exhibited increased antibody titres against sheep red blood cells (SRBC), indicating a stronger humoral response. Similarly, Swain et al. [57] found that dietary supplementation with selenium and vitamin E, whether individually or in combination, significantly enhanced humoral immunity, increasing post-vaccination antibody levels.

In ruminants, selenium plays a critical role in both passive immunity transfer and the stimulation of active immunoglobulin synthesis. Maternal selenium supplementation increases immunoglobulin levels in colostrum and offspring blood, improving resistance to infections [58]. This effect is especially important during the periparturient period, when neonates rely solely on colostrum antibodies. Shojadoost et al. [59] also found that selenium supplementation in chickens reduced viral shedding following avian influenza challenge and upregulated the expression of interferons and antiviral genes, highlighting selenium’s role in enhancing both humoral and innate immunity. Human studies have also reported selenium-dependent improvements in antibody titres and vaccine responses [60]. At the molecular level, selenium’s antioxidant and immunoregulatory functions are closely linked, as selenoproteins such as GPx and TrxR regulate redox balance, B-cell proliferation, and antibody synthesis. No adverse effects were observed during the study, suggesting that SeTG are well tolerated over the short term.

Selenium deficiency remains a common problem in many regions due to low soil selenium levels, leading to impaired immunity, reproductive problems, and metabolic disorders in livestock [1,7,17]. Traditional selenium supplementation strategies rely on inorganic salts (sodium selenite or selenate) or organic forms (selenomethionine, selenium-enriched yeast). Each form has advantages and limitations in terms of bioavailability, retention, and safety [18,61]. The current results suggest that SeTG may represent a promising alternative, potentially offering improved pharmacokinetics and targeted immunomodulatory activity. By improving innate and adaptive immunity, supplementation may reduce susceptibility to infections, support vaccine responses, and decrease reliance on antimicrobial agents in animal production systems.

However, the study has limitations that should be acknowledged. The duration was relatively short (28 days), limiting the ability to assess long-term effects or potential accumulation in tissues. The number of animals was limited, and no challenging experiments were performed to test resistance against pathogens under field conditions. Moreover, mineral interactions, particularly with copper, sulphur, and molybdenum, can strongly influence selenium metabolism and immune function [62,63], and should be considered in future research. Additionally, the possible impact of the lipophilic nature of SeTG on bioavailability and tissue retention warrants exploration.

With this in mind, future research directions can be identified. First, long-term studies are needed to evaluate whether the immunostimulatory effects of SeTG are sustained over time and whether they translate into improved health, reproduction, and productivity outcomes in commercial flocks. Second, comparative trials directly testing SeTG against sodium selenite, selenate, and selenium yeast would clarify relative bioavailability, efficacy, and cost-effectiveness. Third, mechanistic investigations using transcriptomics, proteomics, and metabolomics could elucidate how SeTG influences the expression of selenoproteins, cytokines, and signalling pathways at the cellular and molecular level. Fourth, studies integrating clinical endpoints such as morbidity, mortality, and vaccine responsiveness are essential to validate functional outcomes. Finally, environmental and food safety aspects should be addressed, including selenium excretion, potential residues in edible tissues, and compliance with consumer safety standards.

In summary, dietary supplementation with SeTG enhanced phagocyte activity, lymphocyte proliferation, lysozyme activity as a marker of innate humoral defence, and gamma globulin levels as an indicator of adaptive humoral immunity in sheep, thereby supporting both innate and adaptive immune responses. These findings support the immunomodulatory potential of selenium and demonstrate that SeTG, as a novel selenium compound, may effectively improve immune function. 

## 5. Conclusions

This study demonstrated that dietary supplementation with SeTG, a novel lipophilic selenium compound, enhances selected parameters of innate and adaptive immunity in sheep. Significant improvements were observed in phagocyte activity, lymphocyte proliferation, lysozyme activity, and gamma globulin levels, with no adverse effects on ceruloplasmin activity. These results suggest that SeTG may be a safe and effective alternative to conventional selenium sources in ruminant nutrition.

Further long-term and comparative studies under practical farming conditions are needed to confirm these findings and to determine whether the observed immunomodulatory effects translate into improved health status, productivity, or reduced need for antimicrobial interventions.

## Figures and Tables

**Table 1:** Selected parameters of cellular immunity in sheep after selenitetriglycerides supplementation (mean ± SD).

Parameter	Day	Group		
		Control (C)	Supplemented (S) ^1^	*p*-Value
Respiratory burst activity (OD 620 nm)	0	0.392 ± 0.026	0.439 ± 0.052	0.1104
	14	0.427 ± 0.042	0.505 ± 0.057	0.0108
	28	0.397 ± 0.039	0.600 ± 0.069 ^C^	*p* < 0.0001
Potential killing activity (OD 620 nm)	0	0.394 ± 0.048	0.410 ± 0.051	0.4941
	14	0.393 ± 0.015	0.486 ± 0.038 ^A^	0.0005
	28	0.353 ± 0.030	0.491 ± 0.051 ^A^	*p* < 0.0001
Proliferative response of lymphocytes—ConA	0	1.183 ± 0.185	1.323 ± 0.208	0.7106
	14	2.239 ± 0.806	4.193 ± 1.283 ^C^	*p* < 0.0001
	28	1.792 ± 0.364	2.634 ± 0.134 ^A^	0.0320
Proliferative response of lymphocytes—LPS	0	0.958 ± 0.245	0.933 ± 0.069	0.9179
	14	1.417 ± 0.154	2.390 ± 0.841 ^C^	0.0003
	28	1.151 ± 0.188	1.977 ± 0.464 ^B^	0.0018

SD, standard deviation; numerical results are presented as the arithmetic mean ± SD. ^1^ Different letters within columns indicate strength of the difference within the group (A, *p* ≤ 0.01; B, *p* ≤ 0.001; C, *p* ≤ 0.0001) relative to day 0.

**Table 2:** Selected parameters of humoral immunity in sheep after selenitetriglycerides supplementation (mean ± SD).

Parameter	Day	Group		
		Control (C)	Supplemented (S) ^1^	*p*-Value
Lysozyme activity (mg/L)	0	0.983 ± 0.024	1.005 ± 0.027	0.2538
	14	1.017 ± 0.035	1.067 ± 0.049 ^B^	0.0129
	28	0.991 ± 0.020	1.048 ± 0.033	0.0052
Ceruloplasmin activity (mg/L)	0	386.78 ± 16.977	399.93 ± 13.374	0.2219
	14	394.34 ± 19.832	413.35 ± 20.256	0.0814
	28	407.29 ± 20.698	409.36 ± 17.363	0.8457
Gamma globulin levels (g/L)	0	14.254 ± 1.323	14.915 ± 1.863	0.4564
	14	13.678 ± 1.276	16.139 ± 1.502	0.0086
	28	14.020 ± 1.597	17.146 ± 1.463 ^A^	0.0012

SD, standard deviation; numerical results are presented as the arithmetic mean ± SD. ^1^ Different letters within columns indicate strength of the difference within the group (A, *p* ≤ 0.05; B, *p* ≤ 0.01) relative to day 0.

## Data Availability

All relevant data are contained within the manuscript.
